# Intra-Uterine Therapeutics

**Published:** 1887-09

**Authors:** 


### INTRA-UTERINE THERAPEUTICS.

There is probably no one subject in the whole range of medical practice upon which exists so much difference of opinion among men who are recognized as authorities as upon the subject of intra-uterine therapeutics. One need not be an old man to be able to recall the days when the orthodox treatment of endometritis consisted in the application to the inner surface of the uterus of strong caustic applications, or the insertion into the uterine cavity of pieces of solid nitrate of silver, which were allowed to remain until they gradually melted and disappeared. A few bold practitioners, following the teaching of Lombe Atthill, have even advocated and practiced the application of fuming nitric acid to the endometrium. Even now such methods are not entirely a thing of the past. Pajot, in his remarks before the *Société Obstêtricale et Gynécologique de Paris*, on the tenth of last March, stated that in all cases of endometritis, without regard to their etiology, he still applies nitrate of silver, the strength of the solution varying with the nature of the case. In old, rebellious cases, he even resorts to the actual cautery. He claims that ninety-five out of a hundred cases can be cured by this treatment.

In this country, owing to the dominant influence of the teachings of Emmet, intra-uterine treatment for endometritis has largely gone out of vogue. In his address before the British Medical Association at its annual meeting in 1886, Emmet spoke as follows: "Holding the views I have so long maintained in relation to the cause of the discharges from the uterine canal, it followed that I abandoned early the practice of internal applications, and I believe that I was the first to do so. During the past seven years I have not made, in ordinary practice, an application of iodine within the uterine canal, and had, to a great extent, abandoned the practice sometime before. I may now state that I have long since reached a point in practice where I avoid, if possible, the introduction of any instrument or remedy within the uterus." This teaching has been widely accepted by the American practitioners, although some, like Mundé, for instance, still believe that swabbing out the uterine cavity with Churchill's tincture of iodine constitutes a valuable therapeutic resource. There can be but little doubt that in his peculiar tendency to look upon pelvic cellulitis as the *fons et origo* of all pelvic diseases in women, Emmet has gone to an extreme to which the unbiased observation of intelligent investigators cannot follow him.

In February and March of the present year there appeared in the *Nouvelle Archives d'Obstetrique et de Gynécologie* an elaborate article by Doléris upon "Endometritis and its Treatment," in which this distinguished French gynecologist takes strong ground in favor of the mechanical treatment of endometritis by the curette and by an instrument of his own invention, which he calls the *ecouvillon* or mop. The latter instrument is simply a soft brush at the end of a handle of convenient length. By passing it gently over all portions of the intra-uterine surface, it is claimed that all granulations, new growths, mucus, effete epithelium and decomposing matter of all sorts may be effectually removed without any possibility of injury to the uterine tissues. The instrument seems to be merely a concession to the prejudices of those physicians who cannot believe that the curette is entirely innocuous, since Doléris frankly states that he believes the curette to be equally harmless, and that he usually prefers it in his own practice.

The utility of the blunt curette in the treatment of fungous endometritis with profuse hemorrhage has long been recognized, and it was for this very purpose that the instrument was devised by Thomas. Emmet in America and Schroeder in Germany have been its ardent advocates. But in those varieties of the disease not accompanied by metrorrhagia, Doléris is the first to claim for it especial advantages, and he defends his views with the enthusiasm characteristic of the inventor of a new method of treatment.

It must be evident to every one who has had much gynecological practice that the *ecouvillon* of Doléris is a valuable addition to the medical armamentarium, not only for the treatment of endometritis, but also for the treatment of retained fragments of the ovum after miscarriage. Such fragments are often too small and too numerous to be effectually removed by the curette or the finger, and yet their retention exposes the patient to all the dangers of septic infection. Under such circumstances the *ecouvillon*, sweeping over the whole uterine surface, brushes away all the *débris*, and if followed, as all such intra-uterine manipulation should be, by an antiseptic injection, the danger of septicæmia is evidently reduced to a minimum. In a recent article by Misrachi, sixteen cases are reported in which the *ecouvillon* was used in this way with strikingly favorable results.

From swabbing out the uterus with fuming nitric acid to gently brushing its surface with the *ecouvillon* is a long step, and the contrast between these two methods well illustrates the change which has taken place in gynecological practice in the last quarter century. Nevertheless, we are to-day farther removed from a unanimity of treatment than were our predecessors in the days of Henry Bennett. The reason for this lack of unanimity is found chiefly in the fact that gynecological science has been largely built up by empirical observation. Different observers, working along different and often widely divergent lines, have arrived at seemingly opposite results. An Emmet finds cellulitis where a Lawson Tait finds pyosalpinx. A Doléris advocates the curette where a Pajot uses the actual cautery. The time has not yet arrived when all the facts collected from different sources can be analyzed, compared and sifted and a complete and harmonious system of gynecology deduced therefrom. Until that time we can only go on observing, recording and correcting our data in the full confidence that more extended observation will finally reconcile all seeming discrepancies.

